# Regulation of Metabolic Aging Through Adenosine Mono Phosphate-Activated Protein Kinase and Mammalian Target of Rapamycin: A Comparative Study of Intermittent Fasting Variations in Obese Young Women

**DOI:** 10.3390/nu17101695

**Published:** 2025-05-16

**Authors:** Sheeny Priska Purnomo, Purwo Sri Rejeki, Raden Argarini, Shariff Halim, Dian Aristia Rachmayanti, Chy’as Diuranil Astrid Permataputri, Ivan Kristianto Singgih

**Affiliations:** 1 Master Program of Basic Medical Science, Faculty of Medicine, Universitas Airlangga, Surabaya 60132, East Java, Indonesia or sheenypriska@petra.ac.id (S.P.P.); dr.dianaristia@gmail.com (D.A.R.); chyas.diuranil.fk@upnjatim.ac.id (C.D.A.P.); 2 Faculty of Medicine, Petra Christian University, Surabaya 60236, East Java, Indonesia; 3 Physiology Division, Department of Medical Physiology and Biochemistry, Faculty of Medicine, Universitas Airlangga, Surabaya 60132, East Java, Indonesia; raden-a@fk.unair.ac.id; 4 Faculty of Health Sciences, University Technology MARA (UiTM) Pulau Pinang, Bertam Campus, Kepala Batas 13200, Pulau Pinang, Malaysia; halimshariff@uitm.edu.my; 5 Study Program of Industrial Engineering, University of Surabaya, Surabaya 60293, East Java, Indonesia; ivanksinggih@staff.ubaya.ac.id

**Keywords:** AMPK, mTOR, metabolic age, obesity, intermittent fasting

## Abstract

**Background/Objectives:** Obesity accelerates metabolic aging through oxidative stress, inflammation, and mitochondrial dysfunction. AMP-activated protein kinase (AMPK) and mammalian target of rapamycin (mTOR) are nutrient-sensing pathways regulating metabolism. AMPK promotes energy metabolism and autophagy, while excessive mTOR activity contributes to aging. Intermittent fasting (IF), including time-restricted feeding (TRF)—limiting food intake to a 6 h window (18:6)—and alternate-day modified fasting (ADMF)—alternating 24 h fasting (≤25% daily caloric intake) with unrestricted feeding—may improve metabolic regulation. However, their effects on AMPK, mTOR, and metabolic age remain unclear. **Methods:** This quasi-experimental pre-test–post-test control group study compared the TRF and ADMF on metabolic age, AMPK, and mTOR in young obese women. Twenty-four participants (mean age: 21.29 ± 1.76 years; body fat: 36.92 ± 3.18%; BMI: 29.68 ± 3.70 kg/m^2^) were initially matched by BMI and assigned to Control, TRF, and ADMF groups. A total of 4 participants (1 Control, 3 ADMF) were excluded due to outlier values, yielding final group sizes: Control (*n* = 7), TRF (*n* = 8), and ADMF (*n* = 5). The intervention lasted 20 days. **Results**: A significant decrease in AMPK levels was observed in the ADMF group (*p* = 0.043), while changes in the TRF and Control groups were not significant. mTOR levels showed a decreasing trend but were not statistically significant. No significant changes were found in metabolic age. **Conclusions:** Twenty days of intermittent fasting intervention did not significantly affect AMPK, mTOR, or metabolic age in young obese women. TRF may more effectively enhance AMPK and reduce mTOR, while ADMF may better reduce metabolic age.

## 1. Introduction

Obesity is a multifactorial condition characterized by excessive accumulation of body fat and has become an increasingly prevalent public health issue globally. This condition is usually attributed to an imbalance between energy intake and expenditure, which is reinforced by genetic factors, a high-calorie diet, and low physical activity [1,2,3]. Pathologically, obesity induces dysfunction of adipose tissue, oxidative stress, and low-grade chronic inflammation, all of which are causes of various metabolic disorders, like as insulin resistance, dyslipidemia, and hypertension [4,5]. This condition not only disrupts the physiological balance of the body but also acts as a major risk factor for the development of various chronic diseases, which can ultimately increase morbidity and mortality rates.

Obesity has become an increasingly serious global health crisis, with prevalence continuously rising worldwide, including in middle- and low-income countries. WHO data show that in 2022, there were 2.5 billion adults with overweight, and the global prevalence of obesity has more than doubled between 1990 and 2022 [6]. In Indonesia, the prevalence of obesity among adults has doubled in the past two decades, reaching 21.8% in 2022, with higher rates in urban areas [3]. Women show a higher prevalence of obesity compared to men. According to [7,8], the prevalence of obesity in women aged ≥18 years reached 32.9%, higher than in men at 19.7%. Young women aged 18–24, especially in urban areas, have become a highly vulnerable group due to sedentary lifestyles, high-fat and high-sugar diets, as well as social and academic stress [3]. Early adulthood is a critical period for the maturation of the metabolic system, hormonal stabilization, and the formation of sustainable lifestyle habits. Obesity at this age not only increases the risk of metabolic and reproductive disorders but also accelerates biological aging through mechanisms of chronic inflammation, oxidative stress, and mitochondrial dysfunction [9]. An unhealthy lifestyle at a young age contributes to the decline in cellular function and increases the risk of non-communicable diseases in adulthood [10]. With the increasing number of people with overweight and obesity worldwide, the health burden has also significantly increased [11]. Obesity and age-related diseases are estimated to cost the global economy more than USD 4 trillion by 2035 [12]. This underscores that obesity has evolved into an urgent global health issue that requires special attention, including for vulnerable groups such as young women [2].

Obesity has serious health impacts by accelerating the aging process through multiple biological mechanisms, such as increased oxidative stress, systemic inflammation, and metabolic dysfunction, which all contribute to the amplified state of cellular damage in a similar way to the natural aging process [5]. Research on humans shows that obesity causes dysfunction of adipose tissue, which acts as an endocrine organ and produces pro-inflammatory adipokines such as TNF-α, leptin, and resistin. This imbalance of adipokines can accelerate the aging process by triggering chronic inflammation and metabolic dysfunction. Additionally, individuals with obesity tend to experience insulin resistance and energy homeostasis disturbances, which contribute to accelerated cellular aging [13]. Another study also shows that individuals with obesity tend to experience telomere shortening, which is the end part of chromosomes that plays a role in maintaining genomic stability, potentially accelerating cellular aging and increasing the risk of age-related chronic diseases such as type 2 diabetes, cardiovascular diseases, and neurodegeneration [1]. Additionally, other studies show that the expression of longevity genes such as Forkhead Box O Transcription Factors (FOXO3a) is lower in obese individuals, which can accelerate the aging process by reducing the cells’ ability to cope with oxidative stress and increasing the risk of degenerative diseases [14]. The relationship between obesity and aging not only reduces life expectancy but also shortens the healthy lifespan free from disease [5,15]. Therefore, an approach is needed to immediately reduce the rates of obesity and aging in order to prevent premature aging and reduce the economic and social burden caused by age-related diseases.

Intermittent fasting (IF) has become one of the important strategies in reducing obesity and preventing premature aging. IF includes several main methods, among them Time-Restricted Fasting (TRF), Alternate Modified Day Fasting (ADMF), and the 5:2 Diet. TRF involves restricting daily eating times, such as the 16:8 method, where a person fasts for 16 h and eats within an 8 h window. Meanwhile, ADMF involves fasting on certain days, with very limited calorie intake, and on non-fasting days, one eats normally. The 5:2 diet is an approach where a person undergoes strict calorie restriction for two non-consecutive days in a week, while on the other five days, they eat normally [16]. Physiologically, IF plays a role in improving metabolic health, reducing weight, and lowering the risk of chronic diseases such as type 2 diabetes and cardiovascular diseases [17]. Research on humans shows that IF methods such as time-restricted feeding (TRF) and 5:2 fasting can lead to significant weight loss and improvements in metabolic parameters, such as blood pressure and blood glucose levels, although the effects vary depending on the individual [16]. Additionally, IF is also known to contribute to the improvement of cardiometabolic health by modulating epigenetic pathways and enhancing autophagy, both of which are associated with the prevention of premature aging [18,19]. One of the main mediators in this process is AMPK (AMP-activated protein kinase). AMPK functions as a primary cellular energy sensor or nutrient sensor that regulates energy balance in the body. AMPK is activated in conditions of energy deficit, such as during fasting or physical activity, and plays an important role in fatty acid oxidation, glucose uptake, and suppression of lipogenesis. Additionally, AMPK also plays a role in inducing autophagy, improving mitochondrial function, and reducing oxidative stress, thereby contributing to increased metabolism and slowing the aging process [13,20]. In obesity, AMPK activity decreases, leading to disturbances in energy metabolism [5]. Nevertheless, research on humans regarding the effects of IF on AMPK still shows varied results. Some studies report that IF can increase AMPK activity, but other studies show that this response does not always occur, especially in individuals with obesity or certain metabolic conditions [21]. In fact, several studies have shown that after longer fasting periods, AMPK activity actually decreases or does not change significantly [22]. Furthermore, recent systematic reviews suggest that the metabolic benefits of IF in humans may be more related to overall calorie restriction rather than direct activation of the AMPK pathway [23].

On the other hand, the mTOR (mammalian target of rapamycin) pathway, which is involved in cell growth and protein synthesis, shows an activity pattern opposite to that of AMPK. mTOR is a serine/threonine kinase that plays a crucial role in regulating cell growth, energy metabolism, and protein synthesis. mTORC1 (mTOR Complex 1), one of the main forms of mTOR, is a key component in the regulation of lipid and glucose metabolism and plays an important role in obesity. mTORC1 is activated by excess nutrition, particularly through increased intake of glucose and amino acids, as well as stimulation by insulin. Excessive activation of mTORC1 in obesity contributes to metabolic dysfunction by disrupting lipid metabolism regulation and increasing insulin resistance [4,5,13,24,25]. In obese individuals, mTOR activity increases, resulting in the inhibition of autophagy and excessive anabolic processes that contribute to cellular aging [3,13,20]. A study found that the combination of IF (TRF 16:8) and aerobic exercise can significantly reduce mTOR levels in obese women, which impacts the reduction in inflammation and improvement of cellular metabolism [3]. Additionally, several studies also show that calorie restriction in IF can reduce metabolic age based on epigenetic changes measured through DNA methylation [18]. Because research findings on the effects of fasting on AMPK remain inconsistent—with one study reporting an increase [21], while others show decreases or no significant changes [22]—and given that the impact of different intermittent fasting (IF) regimens on aging-related biomarkers such as AMPK, mTOR, and metabolic age has not been previously compared in obese human subjects, further investigation is warranted. To our knowledge, this is the first study to directly compare time-restricted feeding (TRF) and alternate-day modified fasting (ADMF) in relation to these specific molecular and physiological outcomes, thereby laying important groundwork for future studies.

From the various issues mentioned above, additional research is needed to explore the influence of IF variations as a non-pharmacological strategy in slowing down the cellular aging process through metabolic pathways. This study aims to analyze the effects of two variations of IF, namely time-restricted feeding (TRF) and alternate-day modified fasting (ADMF), on metabolic age, as well as the expression of metabolic biomarkers involved in aging regulation, namely AMP-activated protein kinase (AMPK) and mammalian target of rapamycin (mTOR). The hypothesis proposed in this study is that IF interventions through TRF and ADMF patterns can increase AMPK activity, decrease mTOR activity, and reduce metabolic age in young obese women. The selection of TRF and ADMF is based on previous studies that show both methods have a relatively high adherence rate and provide significant results in weight loss and improvement of metabolic profiles [26,27]. In this study, the TRF used was the 18:6 pattern, which has been scientifically reported to be more effective than the 16:8 pattern in enhancing fat burning and improving metabolism [28]. This quasi-experimental study used a pre-test–post-test control group design, with three groups (control, TRF, and ADMF), and involved young obese female subjects aged 18–25 years with a body mass index (BMI) > 25 kg/m^2^. The results of this study are expected to serve as a basis for the development of metabolic therapy strategies that support a healthy and high-quality aging process.

## 2. Materials and Methods

### 2.1. Study Design

This study was designed as a quasi-experimental investigation employing fasting interventions in humans using a pre-test–pre-test-post-test control group design. Subjects who met the inclusion criteria underwent a matching process based on their BMI for group allocation. Participants with the highest BMI values were ranked in descending order and subsequently assigned to one of three groups: Control (*n* = 8), time-restricted feeding (TRF) (*n* = 8), or alternate-day modified fasting (ADMF) (*n* = 8). The fasting interventions were carried out over a period of 20 days, while the Control group was instructed to maintain their usual dietary habits. Of the initial 24 participants, 4 were excluded from analysis as outliers identified using SPSS. Specifically, 1 participant from the Control group (AMPK measurement) and 3 participants from the ADMF group (2 from mTOR and 1 from AMPK measurements) were excluded due to ELISA results that exceeded the highest standard curve, leaving 20 subjects for statistical analysis.

Anthropometric data, body fat measurements, and blood samples were collected both before (pre-test) and after (post-test) the intervention. If a subject was absent during screening, pre-test, and post-test, or experienced severe illness during the study and could not continue the fasting intervention for at least 80% of the total fasting period, then they were dropped from the study. Pre-test data were gathered prior to initiating the fasting intervention, and post-test data were obtained one day after the final fasting session. All pre-test and post-test measurements were conducted directly by the researchers to ensure consistency and accuracy. Before each measurement, all subjects were instructed to fast for 8 h (with their last meal consumed at 8:00 PM on the previous day until the time of blood sample collection). The measurements were conducted between 07:00 and 10:00 PM. Blood samples were drawn from the median cubital vein and placed in an icebox at approximately 0 °C until they were analyzed for AMPK and mTOR levels using ELISA.

This study has been approved by the Ethics Committee of the Faculty of Medicine, Airlangga University (reference number 36/EC/KEPK/FKUA/2024). All participants were provided with written informed consent before their participation in this study.

### 2.2. Participant Characteristics

The subjects in this study were young, 18–25-year-old obese women selected based on inclusion and exclusion criteria. The inclusion criteria for this study were obese women residing in Surabaya with a body mass index (BMI) classified as obese type 1 and type 2 according to the Asia-Pacific BMI classification (BMI > 25 kg/m^2^), body fat percentage in the high category (>30%), normal fasting blood glucose (FBG) levels and no history of diabetes mellitus, normal hemoglobin (Hb) levels, and normal blood pressure. During the screening process, potential participants were excluded if they had a history of chronic gastrointestinal disorders (chronic gastritis), cardiovascular conditions such as hypertension and atherosclerosis, or any malignancies. Additionally, individuals who reported alcohol consumption, active smoking, or using slimming pills and other routine medications were excluded from this study. The dropout rate in this study was 0%.

### 2.3. Intermittent Fasting Protocol

In this study, two types of intermittent fasting were implemented: time-restricted feeding (TRF) and alternate-day modified fasting (ADMF). TRF was defined as an 18:6 protocol, whereby subjects fasted for 18 consecutive hours and consumed all calories within a 6 h feeding window [29,30]. The fasting period commenced at 20:00 and extended until 14:00 the following day, with the feeding window spanning from 14:00 to 20:00. During the fasting period, subjects were permitted to consume only plain water, devoid of flavorings or sweeteners, while during the feeding period they were allowed to eat ad libitum without any caloric restrictions. This regimen was maintained continuously for 20 consecutive days.

ADMF was defined as a 24 h fasting period during which subjects received less than 25% of their calculated daily energy needs [31,32]—determined by a nutritionist using the Harris–Benedict formula [33] and provided by the researchers via a specialized catering service. On the subsequent day, subjects were allowed unrestricted ad libitum eating. Throughout the intervention phase, subjects were asked to record daily food using an approximated food record method, having received standardized instructions on accurate recording prior to the commencement of the study.

### 2.4. Outcomes Measurement

#### 2.4.1. Body Composition Assessment

During the screening process, hemoglobin, fasting blood glucose, heart rate, and blood pressure were measured. Hemoglobin was measured by finger-prick test using a hemoglobinometer (Accu-Chek Performa; Roche Diabetes Care, Manheim, Germany) while blood glucose was measured using a glucometer (Easy Touch GCU ET322; Zhejiang EasyTouch Medical Instruments Co., Ltd., Zhejiang, China). Heart rate and blood pressure were measured using the digital sphygmomanometer (Omron HEM-8712; Omron Healthcare Co., Ltd., Kyoto, Japan). Body composition was measured using BIA (Omron HBF-375 Karada Scan Body Fat Composition Analyzer; Omron Healthcare Co., Ltd., Japan) [30,34].

#### 2.4.2. Blood Sampling and Biochemical Analysis

A blood sample was taken from the median cubital vein between 07:00 and 10:00 am using a 10 mL Terumo syringe with a 20 G needle. The collected blood was stored in a BD vacutainer SST II Advance Plus Blood Collection Tube. The blood sample was centrifuged at 1000 rpm (5 min, 23 °C) to separate the red blood cells and serum. This procedure is performed within <20 min of blood collection from the subject. The separated serum is used for the analysis of AMPK and mTOR levels. Measurement of AMPK levels using the Human Phosphorylated Adenosine Monophosphate Activated Protein Kinase ELISA kit (catalogue No. E0746Hu, Bioassay Technology Laboratory, Shanghai Korain Biotech Co., Ltd., Shanghai, China) with AMPK sensitivity of 0.28 ng/mL and detection range of 0.5–200 ng/mL [35]. Measurement of mTOR levels using the Human Mammalian Target of Rapamycin ELISA kit (catalogue No. E3693Hu, Bioassay Technology Laboratory, Shanghai Korain Co., Ltd.) with mTOR sensitivity of 0.28 ng/mL and detection range of 0.5–200 ng/mL [36,37].

### 2.5. Statistical Analysis

The sample size was calculated using the software G power version 3.1.9.7. The statistical test selected was an independent-samples *t*-test (difference between two independent means, one-tailed). The analysis assumed an effect size (Cohen’s *d*) of 3.58, derived from AMPK means ± SD of 1.5 ± 0.3 vs. 0.7 ± 0.1 in a previous study [21]. The parameters included a significance level of 0.05 and a statistical power of 80%, and an allocation ratio of 1:1, resulting in a minimum of 3 participants per group. Then, a sample size correction was performed using the Higgins and Kleinbaum formula. According to the formula, the proportion of the sample that dropped out using previous research [3] was found to be *n* = 6 in each group, resulting in a minimum total of 18 subjects across the 3 groups.

All statistical analyses were calculated using IBM SPSS Statistics Software version 27. Normality of the data was examined using the Shapiro-Wilk test. For normally distributed data, paired *t*-tests were used to compare pre-test and post-test means within the same group, while differences between groups were analyzed using one-way analysis of variance (ANOVA), followed by Tukey’s Honest Significant Difference (HSD) post hoc test for pairwise comparisons. For non-normally distributed data, the Wilcoxon signed rank test was used for within-group comparisons, and the Kruskal–Wallis test for between-group comparisons. Data will be presented with mean ± standard deviation (SD) for normally distributed data or median (interquartile range, IQR) for non-normally distributed data. All statistical data analyses used a level of significance at *p* < 0.05.

## 3. Results

### 3.1. Participant Characteristics

The subjects in this study were young, 21.29 ± 1.76-year-old obese women. At baseline, participants were classified as obese with a mean body mass index (BMI) of 29.68 ± 3.70 kg/m^2^, according to the Asia-Pacific classification criteria (BMI > 25 kg/m^2^), and had a high average body fat percentage of 36.92 ± 3.18% (cut-off > 30%). No significant differences were observed among the three groups in terms of age, BMI, heart rate, blood pressure, fasting blood glucose (FBG), fat mass, or visceral fat mass (all *p* > 0.05), indicating comparable baseline characteristics (Table 1). The details of the subject’s characteristics are shown in Table 1.

Before and after weight, BMI, fat mass, and visceral fat mass between groups are shown in Table 2. A downward trend in body weight and BMI was observed in the ADMF group; however, it was not statistically significant (body weight *p* = 0.086; BMI *p* = 0.052).

### 3.2. Effects of Intermittent Fasting on AMPK Levels, mTOR Levels, and Metabolic Age

Table 3 summarizes the comparison of AMPK levels, mTOR levels, and metabolic age between pre-test and post-test for each group. We observed no significant change in the AMPK levels in the Control group (*p* = 0.364). In the ADMF group, AMPK levels significantly decreased (*p* = 0.043). Similarly, the TRF group exhibited a downward trend; however, it was not statistically significant (*p* = 0.744). In the mTOR assessment, a downward trend was observed in all groups; however, it was not statistically significant (*p* > 0.05). Meanwhile, in the metabolic age measurement, the mean values remained stable with no significant differences (*p* > 0.05). In keeping with this, the statistical analysis of changes in AMPK, mTOR, and metabolic age was not significant (Table 4).

**Figure 1:**
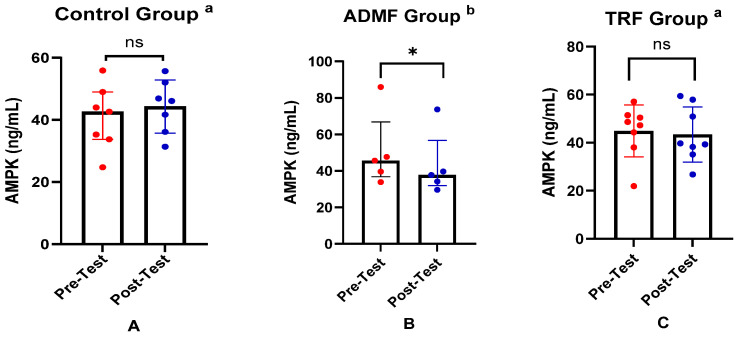
Differences in AMPK levels (ng/mL) between pre-test and post-test in the three groups. (**A**) Control group. (**B**) ADMF group. (**C**) TRF group. ^a^ Representative of data with mean ± SD and *p*-value was obtained by paired sample *t*-test. ^b^ Representative of data with median (interquartile range) and *p*-value was obtained by Wilcoxon signed rank test. (ns) Not significant (*p* ≥ 0.05). (*) Significant at pre-test (*p* ≥ 0.05).

**Figure 2:**
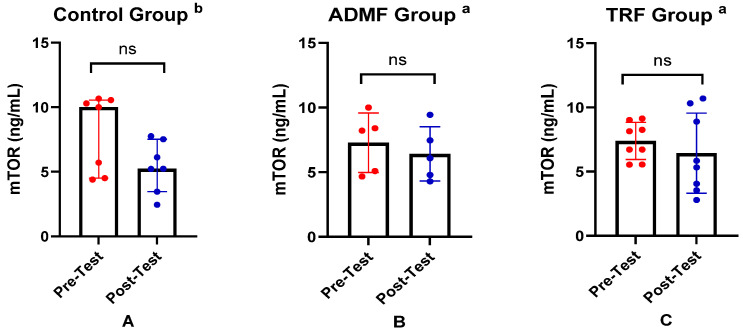
Differences in mTOR levels (ng/mL) between pre-test and post-test in the three groups. (**A**) Control group. (**B**) ADMF group. (**C**) TRF group. ^a^ Representative of data with mean ± SD and *p*-value was obtained by paired sample *t*-test. ^b^ Representative of data with median (interquartile range) and *p*-value was obtained by Wilcoxon signed rank test. (ns) Not significant (*p* ≥ 0.05).

**Figure 3:**
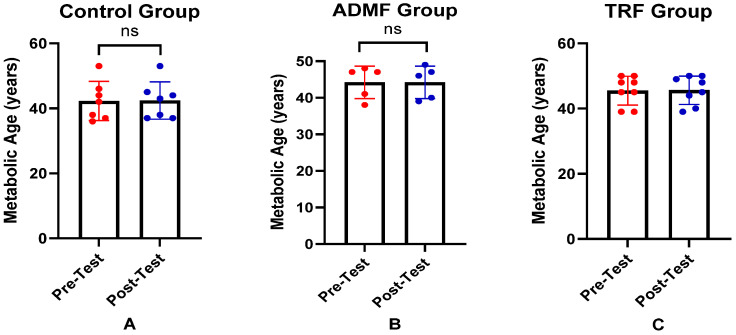
Differences in metabolic age (years) between pre-test and post-test in the three groups. (**A**) Control group. (**B**) ADMF group. (**C**) TRF group. Representative of data with mean ± SD. *p*-value was obtained by paired sample *t*-test. (ns) Not significant (*p* ≥ 0.05).

To facilitate interpretation of the findings, the key differences in biomarker levels and metabolic age are summarized in graphical form. The differences in AMPK levels between pre-test and post-test across the three groups are illustrated in Figure 1. Changes in mTOR levels among the groups are presented in Figure 2, while the differences in metabolic age before and after the intervention in all groups are shown in Figure 3.

## 4. Discussion

The results of the analysis of the subject characteristics data show that the three groups are homogeneous in terms of age, BMI, baseline AMPK, mTOR, metabolic age, and other clinical parameters. This indicates that the three groups had controlled variables, allowing for a more accurate interpretation of the results, particularly in comparing the impact of each fasting variation on molecular biomarkers and metabolic age.

Despite this rigor in design, studies focusing exclusively on young obese women remain scarce, which limits direct comparisons with previous IF trials and underscores the novelty of our work in elucidating AMPK/mTOR modulation and metabolic age changes in this specific demographic.

AMPK-level analysis showed a significant decrease in the ADMF group and a decreasing trend in the TRF group, while the Control group experienced an increase. These results are consistent with previous studies [21,23], which show that fasting does not always consistently activate AMPK, especially in obese individuals. These findings indicate that in individuals with obesity, the metabolic adaptation mechanisms to TRF are likely not mediated by direct activation of the AMPK pathway, but rather through improvements in other metabolic aspects related to reduced calorie intake.

In ADMF, the decrease in AMPK that does not align with the initial hypothesis may be caused by the refeeding phase, the duration and pattern of fasting, and the metabolic status of the subjects [18,38]. The refeeding phase causes the body to switch from a catabolic state to an anabolic state [38]. The insulin spike that occurs during refeeding suppresses AMPK activity through the activation of the PI3K-Akt-mTOR pathway, which is an antagonist of AMPK [20,22]. Nutritional composition also plays a role, where high carbohydrate consumption inhibits the activation of LKB1, an upstream regulator of AMPK [20], while a low-carbohydrate or high-fat diet can support AMPK through ketogenesis [18,20,39]. Non-fasting days on ADMF with ad libitum intake contribute to insulin spikes, increased mTOR activity, and AMPK suppression. TRF shows more controlled metabolic fluctuations due to having a more limited eating window, so the insulin spike is not as strong as in ADMF. The difference in refeeding patterns affects the stability of AMPK activation.

Hormonal factors such as cortisol and IGF-1 also play a role. In ADMF, a more fluctuating eating pattern with alternating fasting and refeeding periods can lead to higher cortisol levels compared to TRF. High cortisol can inhibit AMPK through the activation of SGK1 (serum- and glucocorticoid-regulated kinase-1), which phosphorylates AMPK at its inhibitory residue and suppresses its activity [21,40]. IGF-1 increases during the refeeding phase due to a surge in insulin and higher protein intake. Meanwhile, the increase in IGF-1 can also activate the PI3K-Akt-mTOR pathway, which acts as an inhibitor of AMPK [20]. In ADMF, a longer refeeding period compared to TRF can significantly increase IGF-1, which contributes to a stronger inhibition of AMPK. On the other hand, in TRF, the increase in IGF-1 is more moderate because refeeding occurs within a more limited time window, resulting in a smaller suppressive effect on AMPK.

A fasting duration that is not long enough can limit AMPK activation. To ensure stable AMPK activation in the body, a consistent adaptation period of intermittent fasting duration over several weeks is needed to create sustainable metabolic adaptation. If the fasting period in the ADMF pattern is not long enough, the cells may not reach the necessary level of metabolic stress to optimally activate AMPK [18,20,40]. The same goes for TRF, where the effects on AMPK are not as pronounced compared to stricter IF, because TRF allows calorie consumption within a certain time frame, which can reduce the potential for significant energy depletion and lead to more subtle metabolic adaptations to significantly activate AMPK. Metabolic adaptation requires an intervention of at least 2–6 weeks. TRF with 16 h of fasting per day only shows consistent AMPK activation after 2–4 weeks [18,24,40,41]. This is due to increased autophagy, insulin sensitivity, and reduced insulin levels resulting from repeated metabolic stress occurring during fasting periods. Other studies also note that in alternate-day modified fasting (ADMF), significant AMPK activation is observed after 4–6 weeks, with the additional effect of increased lipid metabolism supporting long-term energy efficiency [20,40,42].

After understanding the role and dynamics of AMPK in responding to intermittent fasting interventions, we will next examine the response of the mTOR pathway as the main metabolic regulatory mechanism. mTOR analysis showed a non-significant decreasing trend, unlike a previous study [3] that used TRF 16:8 for 2 weeks (five times a week) in young obese women with an ad libitum diet during the refeeding phase, which showed a significant decrease in mTOR. This difference may be due to the different fasting and refeeding patterns. According to [43], a structured refeeding phase—with a longer eating window (e.g., 8–12 h) and non-fasting days—can prevent overcompensation, a condition where excessive calorie intake during the eating phase repeatedly causes insulin spikes and reactivation of the mTOR pathway. In this research, a shorter eating window (6 h) and daily fasting without breaks allowed for the accumulation of refeeding effects, causing repeated insulin spikes that activated mTOR; thus, the mTOR inhibition effect was not significantly observed. Additionally, another study [44] also supports that an unstructured refeeding phase can diminish the metabolic adaptation benefits of fasting. Therefore, the impact of IF on mTOR highly depends on the duration of fasting, the intensity of the refeeding phase, and the balance between the fasting and eating phases [32,45,46]. In the TRF 18:6 protocol, the 6 h eating phase provides an opportunity for nutrient intake that can increase amino acid and insulin levels, which subsequently reactivates mTOR. Thus, although mTOR is inhibited during the fasting phase, this effect can be compensated by the refeeding period, especially if the food consumed after fasting is high in protein or carbohydrates. Proteins, especially amino acids like leucine, are known to be direct activators of mTOR. If the respondents in this study still consume a high amount of protein during the eating window, then the mTOR inhibition effect that occurs during the fasting phase could be quickly compensated by the reactivation of mTOR during the eating phase [39,47]. Therefore, the differences that are found are not significant enough to be statistically significant [11,44,48]. Thus, although both studies used the TRF approach, differences in the duration of the eating window and refeeding patterns were key factors influencing the significance of mTOR reduction.

In addition, the relatively short duration of the intervention, which was only 20 days, is likely also a contributing factor. The mTOR pathway is highly sensitive to nutrient availability and metabolic signals, so significant changes in mTOR activity require longer metabolic adaptation. Studies on Alternate Day Fasting (ADF) show that metabolic adjustments affecting the mTOR pathway become more apparent after ≥4 weeks of intervention [49]. This is consistent with the findings of [45,50], who state that the adaptive effects of intermittent fasting—both TRF and ADMF—especially on the mTOR pathway, generally only become apparent after a minimum of 4–8 weeks. The study by [14], which used a 10-day periodic fasting protocol on healthy individuals, also showed that mTOR protein expression did not significantly decrease, although there was an increase in the expression of longevity protection genes such as SIRT1 and FOXO3. This further emphasizes that to significantly reduce mTOR activity, a longer metabolic adaptation period is required. Therefore, a longer intervention duration and a more structured refeeding approach are needed to achieve significant mTOR suppression effects [45,46,48].

A study by [39] showed that the effects of time-restricted feeding (TRF) on mTOR expression also depend on meal timing. If the eating schedule is not aligned with the body’s circadian rhythm—such as eating more in the evening—then the mTOR suppression effect might be weaker compared to interventions that optimize meal timing in the morning to afternoon. In the eTRF (early TRF) study, eating within the morning window (08:00–14:00) is more effective in regulating metabolism compared to eating later in the evening. Therefore, although the 18:6 TRF protocol in this study allows for a sufficiently long fasting phase, the misalignment of refeeding time with the circadian rhythm, as well as the possibility of high protein or carbohydrate consumption during the eating window, may reduce the expected mTOR inhibition effect.

Subsequent to the analysis of mTOR activity alterations, the following step is to examine its influence on metabolic age, a crucial metric for evaluating metabolic adaptation and overall biological health. It is evident from the results that the intermittent fasting intervention in this study did not produce significant differences in metabolic age between the control, TRF, and ADMF groups, although there was a trend of decreasing BMI and body weight in the ADMF group. The 20-day intervention duration is likely not sufficient to induce significant changes in metabolic biomarkers that reflect biological age. Metabolic adaptations, such as increased insulin sensitivity, improved lipid profiles, and reduced inflammation, only become apparent after a longer duration of intervention [51], Thus, it can be claimed that gradual adaptation is necessary for metabolic improvement [15,32].

Furthermore, fat mass as an indicator of metabolic age did not experience significant changes in this study, despite a trend of weight loss and BMI reduction in the ADMF group. This is different from the previous research [52], who reported a significant reduction in fat mass (~9%) in the TRF intervention combined with exercise over 12 weeks. This comparison suggests that a minimum intervention duration of 12 weeks, along with the addition of moderate to high physical activity, may be necessary to achieve optimal metabolic adaptations.

Several studies support that a longer intervention duration, at least around 12 weeks, is necessary to induce significant changes in metabolic biomarkers. For example, refs. [17,53] reported that a 12-week (3-month) TRF intervention in obese individuals resulted in improvements in blood pressure, lipid profile, fat mass, and other metabolic parameters that contribute to a reduced risk of metabolic diseases and premature aging. Another study [54] also suggests that the metabolic adaptations underlying the reduction in biological age tend to occur after a sufficiently long intervention period; therefore, at least 3 months may be needed to see meaningful changes. In contrast, an 8-week 5:2 intermittent fasting trial among obese female employees in Jakarta yielded only modest weight loss (−0.8 kg) and BMI reduction (−0.3 kg/m^2^), without any significant changes in fat mass or fat-free mass [55]. The optimal duration can vary depending on the initial condition of the subject, adherence to fasting protocols, and other individual factors. Similar findings were also obtained by [29], who in a 12-week study on early time-restricted feeding (eTRF) found increased insulin sensitivity and improvement in metabolic circadian rhythm. Both of these factors are crucial in assessing metabolic age because increased insulin sensitivity can prevent insulin resistance, while improved circadian rhythm helps optimize overall metabolism.

Furthermore, ref. [50] in their review on the effects of intermittent fasting concluded that fundamental metabolic adaptations, which include improvements in aging biomarkers such as glucose metabolism, lipids, and inflammation, are generally only observed after a long intervention period, namely more than 12 weeks. This opinion is supported by evidence that structural and functional metabolic adaptations require sufficient time for cells and tissues to optimize their response to fasting and eating cycles. Thus, although IF intervention can theoretically improve metabolic health and reduce biological age, existing evidence shows that the short duration of the intervention—as in this study, namely 20 days—is not sufficient to produce significant changes. Therefore, subsequent research is recommended to use an intervention duration of at least 12 weeks so that changes in metabolic age can be detected more clearly and provide a more comprehensive understanding of the underlying metabolic adaptation mechanisms that support the benefits of intermittent fasting [17,29,50,52,53].

This study has several limitations that should be considered when interpreting the results. First, the lack of strict control over calorie intake and macronutrient composition during the refeeding phase in the intervention group may have influenced the AMPK and mTOR metabolic pathways, potentially obscuring the intended effects of intermittent fasting. Second, variations in participants’ home environments—since all interventions were conducted in free-living conditions—made it difficult to ensure adherence to the fasting protocols. The absence of environmental standardization may have introduced additional variability, potentially affecting internal validity. Furthermore, the 20-day intervention period may have been insufficient to induce significant metabolic adaptations. Future studies should consider extending the intervention duration to at least 3 months to allow physiological processes to fully respond to dietary changes. Incorporating serial measurements throughout the intervention could also enable better monitoring of metabolic improvements over time and provide more comprehensive insights into the long-term effects of intermittent fasting. Moreover, conducting studies in more controlled settings, such as dormitories or clinical research centers, will reduce environmental variability and enable direct monitoring of participants’ dietary intake to strictly control calorie intake and macronutrient composition during refeeding. While the current findings offer valuable initial insights, the small sample size limits generalizability. Larger, well-controlled trials are necessary to validate these results and explore inter-individual variability in response.

## 5. Conclusions

The conclusion of this study is that a 20-day intermittent fasting intervention does not significantly increase AMPK levels nor decrease mTOR expression and metabolic age in young obese women. Although the results did not show statistically significant differences, time-restricted feeding (TRF) demonstrated a more favorable tendency in increasing AMPK activity and reducing mTOR compared to alternate-day modified fasting (ADMF). However, in reducing metabolic age—as reflected by changes in body weight, fat mass, and visceral fat—ADMF appeared to be more effective than TRF. These findings are specific to young obese women and may not be generalizable to other populations. Further studies involving strict dietary control during refeeding, extended intervention durations to allow sufficient metabolic adaptation, and serial measurements to capture temporal dynamics of metabolic responses are recommended.

Despite the limited sample size, this study provides preliminary evidence and novel comparative insight between two IF patterns on key molecular markers of aging. The findings lay a foundation for future research on personalized fasting interventions targeting metabolic and cellular health.

## Figures and Tables

**Table 1:** Participants’ baseline characteristics.

	Control (*n* = 7)	ADMF (*n* = 5)	TRF (*n* = 8)	*p* Value
Age, years ^a^	20.60 ± 1.82	21.20 ± 2.28	20.4 ± 1.14	0.877
Blood pressure, mmHg				
Sistolic ^a^	114.40 ± 10.26	110.60 ± 8.65	122.20 ± 15.88	0.185
Diastolic ^a^	80.20 ± 8.32	77.60 ± 2.70	84.40 ± 11.41	0.471
Fasting Blood Glucose, mg/dL ^b^	101 (98–139.5)	102 (92–120)	95 (88.5–118.5)	0.878
Haemoglobin, mg/dL ^a^	12.46 ± 1.54	13.2 ± 2.41	12.80 ± 1.39	0.920
Weight, kg ^a^	76.49 ± 12.03	73.46 ± 11.06	79.19 ± 5.73	0.591
Height, cm ^a^	157.80 ± 6.51	155.7 ± 6.91	161.30 ± 2.71	0.397
BMI, kg/m^2 a^	30.58 ± 5.25	30.08 ± 3.44	30.48 ± 2.28	0.824
Fat mass, % ^a^	37.16 ± 3.86	36.86 ± 4.69	37.58 ± 2.57	0.880
Visceral Fat, % ^b^	9 (7.25–16)	9.5 (7.75–13)	10 (8.25–12)	0.319
AMPK, ng/mL ^b^	35.29 (29.22–43.30)	45.64 (36.75–66.83)	44.29 (29.97–50.92)	0.546
mTOR, ng/mL ^b^	1029 (7.25–10.61)	8.22 (4.89–9.21)	8.14 (6.72–9.07)	0.751
Metabolic age, years ^a^	44.20 ± 6.18	44.20 ± 4.44	45.40 ± 4.16	0.484

Description: ADMF, alternate-day modified fasting; TRF, time-restricted feeding; BMI, body mass index; AMPK, AMP-activated protein Kinase; mTOR, mammalian target of rapamycin. Representative of data with mean ± SD or median (IQR1-IQR3). ^a^ *p*-value was obtained by One-Way ANOVA. ^b^ *p*-value was obtained by Kruskal–Wallis.

**Table 2:** Before and after weight, BMI, fat mass, and visceral fat mass.

Parameter	Group	Pre-Test	Post-Test	*p*-Value
Weight, kg	Control (*n* = 7) ^a^	76.49 ± 12.03	76.25 ± 11.87	0.656
	ADMF (*n* = 5) ^a^	73.46 ± 11.06	72.38 ± 10.82	0.086
	TRF (*n* = 8) ^a^	79.19 ± 5.73	79.57 ± 5.24	0.877
BMI, kg/m^2^	Control (*n* = 7) ^b^	27.10 (25.10–29.78)	27.55 (25.1–29.1)	0.786
	ADMF (*n* = 5) ^a^	30.38 ± 3.52	29.94 ± 3.61	0.052
	TRF (*n* = 8) ^a^	30.14 ± 2.54	30.21 ± 2.58	0.351
Fat Mass, %	Control (*n* = 7) ^a^	37.16 ± 3.86	37.34 ± 3.82	0.907
	ADMF (*n* = 5) ^a^	36.86 ± 4.69	37.26 ± 3.87	0.698
	TRF (*n* = 8) ^a^	37.58 ± 2.57	37.58 ± 2.42	0.464
Visceral Fat, %	Control (*n* = 7) ^b^	9 (7.25–16)	9 (7.25–15.5)	0.655
	ADMF (*n* = 5) ^a^	10.20 ± 2.73	9.10 ± 3.05	0.605
	TRF (*n* = 8) ^a^	10.13 ± 2.25	10.19 ± 3.58	0.961

Description: ADMF, alternate-day modified fasting; TRF, time-restricted feeding; BMI, body mass index. Representative of data with mean ± SD or median (IQR1-IQR3). ^a^ *p*-value was obtained by One-Way ANOVA. ^b^ *p*-value was obtained by Kruskal–Wallis.

**Table 3:** Comparison of AMPK levels, mTOR levels, and metabolic age between pre-test and post-test for each group.

Variable	Group	Pre-Test	Post-Test	*p*-Value
AMPK levels, ng/mL	Control (*n* = 7) ^a^	36.07 ± 7.75	41.63 ± 8.24	0.364
	ADMF (*n* = 5) ^b^	45.64 (36.75–66.83)	37.81 (31.95–56.71)	0.043 *
	TRF (*n* = 8) ^a^	41.22 ± 12.05	39.45 ± 11.42	0.744
mTOR levels, ng/mL	Control (*n* = 7) ^b^	10.29 (7.25–10.61)	6.12 (2.94–7.64)	0.128
	ADMF (*n* = 5) ^a^	7.28 ± 2.3	6.42 ± 2.1	0.621
	TRF (*n* = 8) ^a^	7.94 ± 1.18	4.32 ± 1.26	0.499
Metabolic age, ng/mL	Control (*n* = 7) ^a^	44.20 ± 6.18	44.40 ± 5.73	0.604
	ADMF (*n* = 5) ^a^	44.20 ± 4.44	44.20 ± 4.44	1.000
	TRF (*n* = 8) ^a^	45.40 ± 4.16	45.40 ± 3.85	0.598

Description: ADMF, alternate-day modified fasting; TRF, time-restricted feeding; AMPK, AMP-activated protein kinase; mTOR, mammalian target of rapamycin. Representative of data with mean ± SD or median (IQR1-IQR3). * significant at Control, ADMF, or TRF (*p<* 0.05). ^a^ *p*-value was obtained by paired t-test. ^b^ *p*-value was obtained by Wilcoxon sum rank test.

**Table 4:** Comparison analysis of changes in AMPK, mTOR levels, and metabolic age before and after intervention in the three groups.

Group	Δ AMPK, ng/mL ^b^	*p* Value	Δ mTOR, ng/mL ^a^	*p* Value	Δ Metabolic Age, Years ^a^	*p* Value
Control (*n* = 7)	5.56 ± 10.79	0.174	−3.75 ± 3.68	0.618	0.20 ± 0.84	0.943
ADMF (*n* = 5)	−7.54 ± 4.49		−0.86 ± 3.61		0.00 ± 1.00	
TRF (*n* = 8)	−1.76 ± 13.83		−3.63 ± 0.43		0.00 ± 0.70	

Description: ADMF, alternate-day modified fasting; TRF, time-restricted feeding; AMPK, AMP-activated protein kinase; mTOR, mammalian target of rapamycin. Representative of data with mean ± SD. ^a^ *p*-value was obtained by One-Way ANOVA. ^b^ *p*-value was obtained by Kruskal–Wallis.

## Data Availability

The data presented in this study are available upon request from the corresponding author. The data are not publicly available due to ethical reasons.
